# Grazing Mares on Pasture with Sycamore Maples: A Potential Threat to Suckling Foals and Food Safety through Milk Contamination

**DOI:** 10.3390/ani11010087

**Published:** 2021-01-05

**Authors:** Benoît Renaud, Anne-Christine François, François Boemer, Caroline Kruse, David Stern, Amandine Piot, Thierry Petitjean, Pascal Gustin, Dominique-Marie Votion

**Affiliations:** 1 Department of Functional Sciences, Pharmacology and Toxicology, Fundamental and Applied Research for Animals & Health (FARAH), Faculty of Veterinary Medicine, University of Liège, 4000 Liège, Belgium; acfrancois@uliege.be (A.-C.F.); p.gustin@uliege.be (P.G.); 2 Biochemical Genetics Laboratory, CHU Sart Tilman, University of Liège, 4000 Liège, Belgium; f.boemer@chuliege.be (F.B.); amandine.piot@chuliege.be (A.P.); 3 Department of Functional Sciences, Physiology and Sport Medicine, Fundamental and Applied Research for Animals & Health (FARAH), Faculty of Veterinary Medicine, University of Liège, 4000 Liège, Belgium; caroline.kruse@uliege.be; 4 Equine Pole, Fundamental and Applied Research for Animals & Health (FARAH), Faculty of Veterinary Medicine, University of Liège, 4000 Liège, Belgium; d.stern@uliege.be (D.S.); dominique.votion@uliege.be (D.-M.V.); 5 Association Régionale de Santé et d’Identification Animales (ARSIA—ASBL), Animal Health Department, 5590 Ciney, Belgium; thierry.petitjean@arsia.be

**Keywords:** equine atypical myopathy, *Acer* spp., milk, toxins, environment

## Abstract

**Simple Summary:**

Equine atypical myopathy is seasonal poisoning resulting from the ingestion of seeds and seedlings of the sycamore maple that contains toxins. Literature mentions several cases of intoxication among gravid mares and in unweaned foals. The objective of this study was to determine whether the toxins responsible for atypical myopathy could pass to the foal via suckling. Four mares that were pasturing with sycamore in the vicinity were milked. Analysis revealed the presence of toxins in milk. This unprecedented observation could partially explain cases of unweaned foals suffering from atypical myopathy. However, a transplacental transfer of the toxin cannot be excluded for newborn cases. Besides being a source of contamination for offspring, milk contamination by toxins from fruits of trees of the *Sapindaceae* family might constitute a potential risk for food safety regarding other species’ raw milk or dairy products.

**Abstract:**

Equine atypical myopathy (AM) is seasonal intoxication resulting from the ingestion of seeds and seedlings of the sycamore maple (*Acer pseudoplatanus*) that contain toxins, among them, hypoglycin A (HGA). Literature mentions several cases of AM among gravid mares and in unweaned foals. The objective of this study was to determine whether HGA and/or its metabolite are present in milk from grazing mares exposed to sycamore maple trees as confirmed by detection of HGA and its metabolite in their blood. Four mare/foal couples were included in the study. Both HGA and its metabolite were detectable in all but one of the milk samples. To our knowledge, this is the first study describing transfer of HGA to the milk. This unprecedented observation could partially explain cases of unweaned foals suffering from AM. However, a transplacental transfer of the toxin cannot be excluded for newborn foals. Besides being a source of contamination for offspring, milk contamination by toxins from fruits of trees of the *Sapindaceae* family might constitute a potential risk for food safety regarding other species’ raw milk or dairy products.

## 1. Introduction

Atypical myopathy (AM) is a non-exercise-induced rhabdomyolysis syndrome that strikes grazing equids on a seasonal rhythm. This condition has been initially described in the 1980s in the United Kingdom [1], but was later reported in about 15 European countries [2], in the US [3], and in New Zealand [4].

It was first discovered that AM occurs following ingestion of the toxin hypoglycin A (HGA) mainly present in seeds [5] and seedlings [6] of some species of the *Acer* genus. In Europe, the sycamore maple (*Acer pseudoplatanus)* is the main incriminated species [5,7]. However, in North America, the main threat is the box elder (*Acer negundo*) [3]. In 2019, a second toxin, methylenecyclopropylglycine (MCPG), has been described as playing a part in the ethiopathogenicity of AM [8]. This toxin is a structural analog of HGA, and is also present in sycamore maple seeds [9]. To our knowledge, MCPG has not been sought in seedlings but might also be present.

The active metabolites of HGA and MCPG that are, respectively, methylenecyclopropylacetyl-CoA (MCPA-CoA), and methylenecyclopropylformyl-CoA (MCPF-CoA), are potent inhibitors of enzymes participating in β-oxidation and thus energy production from lipid metabolism [10]. This results in severe energetic impairment in oxidative muscles such as postural, respiratory muscles, and the myocardium [11]. The metabolic disturbances induced by the toxins result in increased concentrations of blood acylcarnitines [12] to an extend that reflects the severity of the toxic process [13]. Both HGA and MCPG, as well as their toxic metabolites, conjugated to carnitine or glycine have been detected in the blood or urine of affected horses [8,14].

It has been previously published that HGA can be detected in the serum of unaffected co-grazing horses [6] while its metabolite, MCPA-carnitine, was found at either very low levels or below the detection limit [8] in the serum of these healthy co-grazers. This confirms the existence of subclinical cases [15].

Literature mentions several cases of AM among gravid mares and in unweaned foals [2]. In 2018, Karlíková et al. reported a newborn foal with signs of AM [16]. This case-report describes a foal born at full term from a mare which recovered from AM during the sixth month of pregnancy. The diagnosis of AM for the foal and the mare resulted from a combination of compatible clinical signs and results from analyses of the foal’s blood with a severely increased activity of the serum creatine kinase (CK), presence of MCPA-carnitine and increased concentration of acylcarnitines. Neither the mare nor the foal was tested for HGA. Authors of this case-report suggest two hypotheses to explain that this newborn foal was affected by AM. The first hypothesis points to a possible excretion of HGA or its metabolites into the mare’s milk and the second one to a transplacental toxin transfer.

The objective of this study was to determine whether HGA or MCPA-carnitine are present in milk from mares exposed to sycamore maple trees.

## 2. Materials and Methods

### 2.1. Animal Selection

From autumn 2018 to spring 2019, blood samples were collected at admission from every horse with a tentative diagnosis of AM at the referral equine hospital of the University of Liège (CVU-ULiège). Whenever possible, blood samples were taken from their equine co-grazers, at pasture, within 24 h of admission of the AM case. When unweaned foals were present, blood and milk from the mares were sampled too.

The Animal Ethics Committee of the University of Liege was consulted. All procedures of this study are in accordance with the national and international guidelines for animal welfare. Since the whole procedure is part of routine veterinary practice to establish a diagnosis or to prevent AM, an agreement number was not required. Owners gave informed consent for their horses’ inclusion in the study.

Among the suspected cases of AM or healthy cograzers, four mare/foal pairs were included in the study. All of the pairs had been exposed to sycamore maple trees on pastures located in Belgium. For the selected cases, age, breed, gender and date of sampling were collected (Table 1). Foals 1, 3, and 4 were referred to the CVU-ULiège with a suspicion of AM. Following a diagnostic algorithm for AM, that has been previously described and used in other [2], only foal 4 was confirmed as AM. Indeed, foal 4 was the only one presenting both pigmenturia and a serum creatine kinase activity over 10,000 UI/L (Table 2). The tentative diagnosis of AM has been corrected into enterocolitis for foals 1 and *Rhodococcus equi* infection for foal 3. Foal 2 and mare 2 were sampled on the field as healthy cograzers of a horse confirmed for AM at the CVU-ULiège.

### 2.2. Blood and Milk Sampling

Whole blood was collected by jugular venipuncture and from each mare, 3 to 5 mL of milk were obtained by hand milking. Collected blood and milk were kept at 4 °C before to be aliquoted within the hour and stored at −80 °C up to analysis.

It is of note that for reasons beyond our control, milk and blood sampling in mare 1 was performed four days after the mare returned to the stable and not during admission with its foal.

### 2.3. Hypoglycin A Assay

HGA in serum was performed according to a previously described protocol [17]. Quantification of HGA was carried out using a TRAQ^®^ kit for amino acid analysis of physiological fluids (Sciex, Farmingham, MA, USA). Samples’ HGA was derivatized using an isotopic tag (mass *m*/*z* 121), while a second labeling reagent (mass *m*/*z* 113) allowed absolute quantification. Derivatized samples were introduced into a TQ5500 tandem mass spectrometer (Sciex, Farmingham, MA, USA) using a Prominence AR HPLC system (Shimadzu, Kyoto, Japan). This method presented a lower limit of quantification of 0.090 μmol/L [17].

To improve method sensitivity on milk’s sample, milk’s HGA was assessed using a slightly modified protocol. Briefly, we increased the initial sample volume (40 > 80 µL) and the injection volume into the analytical system (10 > 25 µL).

### 2.4. MCPA-Carnitine Determination Method

MCPA-carnitine separation and determination were carried out by ultra-performance liquid chromatography combined with subsequent mass spectrometry (UPLC-MS/MS) as reported by Valberg et al. in 2013. The corresponding limit of detection is approximately 0.001 nmol/L [3].

## 3. Results

The selected cases were from various breeds: paint horse, quarter horse, Zangersheide, and light draft horse. The foals were 1 to 5-months old, which implies that none of them was exclusively milk-fed at the time of sampling (Table 1).

Hypoglycin A was detectable in milk samples of mares 1, 3 and 4. In all but one milk sample (mare 2), MCPA-carnitine was found at a concentration above 2 nmol/L (Table 2).

Mares 2, 3, and 4 had a measurable amount of HGA in their blood (Table 2). This observation fits with their exposure to sycamore maple. No HGA was detected in the blood of mare 1. Despite none of the mares showing clinicals signs, all mares presented MCPA-carnitine in their blood. The detected concentrations of MCPA-carnitine ranged from 0.35 nmol/L to 8.16 nmol/L.

All four foals had HGA and MCPA-carnitine in their blood but foal 4 presented a serum MCPA-carnitine concentration several times higher than the others.

## 4. Discussion

To our knowledge, this is the first study to describe HGA and MCPA-carnitine transfer to the milk from mares that are exposed to seedlings or samaras of the sycamore maples.

On the one hand, HGA concentrations measured in milk (mean ± SD = 2.90 × 10^−2^ ± 2.57 × 10^−2^ µmol/kg, *n* = 3) are far smaller than the HGA concentration in autumnal sycamore maple seeds in Europe as reported by the literature [6]. Nevertheless, the question of foals’ sensitivity to HGA is unknown. In 2006, Blake and collaborators described a maximum tolerated dose (MTD) of HGA for rats. Using a repeated dosing toxicity over 30 days, the MTD in feed was 1.50 mg HGA/kg/day [18]. Above this dose, rats presented appreciable organ dysfunction, reduction in life span or at least 10% retardation of body weight gain in growing animals. Extrapolation of this dose to a 40 kg foal, without allometric scaling, yields a maximum tolerated HGA dose of 60 mg/foal/day. This hypothetic MTD would correspond to the unlikely quantity of more than 14,000 L of milk per foal and per day. These figures are highly speculative: foals may be impacted by HGA toxicity very different levels from rats or adult horses. Also, while being an acute disorder, AM appears following several days of exposure to the toxins [19] suggesting an accumulation of toxins before the onset of clinical signs. Therefore, the MTD based on the daily consumption of milk may not reflect the actual risk of intoxication. On the other hand, the MCPA-carnitine concentrations measured in milk (0.00 to 9.47 nmol/L) are close to the minimal observation of 0.96 nmol/L quantified in serum of a horse affected by AM [7].

In this study, all four foals were unweaned but too old to be exclusively milk-fed. The majority of the HGA detected in the blood most likely originates from ingested sycamore samaras/seedlings. However, HGA milk contamination takes part in the toxic pressure. Both HGA and MCPA-carnitine transfer through milk could contribute to cases of unweaned foals suffering from AM. This brings out a new risk factor associated with grazing lactating mares and/or milk banking. Besides, in newborn foals, a transplacental transfer of the toxins could contribute to the risk of AM.

It should be noted that the second incriminated toxin MCPG and its metabolite (i.e., MCPF) might transfer to milk too.

Other toxins present in feed are incriminated in milk contamination in various species, including horses. Multiple publications exist about mycotoxins in milk [20]. Aflatoxins B1, G1, and M1 are reported in milk from lactating animals that ingest contaminated feed [21]. As it can be expected for HGA, aflatoxin concentration in milk changes during the year, following the seasonal pattern of the mother intakes [22]. In addition to raw milk, aflatoxins were found in milk products like cheese and yogurts. One of the toxin responsible for AM—i.e., HGA—was described as heat resistant in aqueous solution for half an hour at 60 °C [23] and stable over time in dry vegetal matrix [24]. Therefore, HGA could also represent a threat to food safety.

Even if in western Europe, horse and donkey milk consumption is currently expanding due to their supposed health-enhancing properties [25], HGA contaminated mare’s milk seems unlikely to trigger an acute intoxication. However, low-dose and long-term exposure to HGA has never been investigated. Unpublished data also reveals that HGA and MCPA-carnitine can be found in blood from multiple ruminant species. Therefore, contamination of raw milk and milk products from other milk-producing species such as cows, sheep, and goats may be problematic. The search for toxins in milk from small and large ruminants exposed to sycamore maples is required to evaluate the potential risk.

Human exposure to HGA and MCPG is already related to worldwide illness-outbreaks through the ingestion of fruits from the *Sapindaceae* family [26,27]. It should be noted that sycamore maple belongs to the same family. Two diseases are reported, each of them with a different symptomatology. The first one, the Jamaican vomiting sickness, is associated with the consumption of unripe akee fruit [26]. The second one is an acute toxic encephalopathy which is linked to litchi fruit [28]. In regions where these diseases are endemic, ingestion of fruits from trees of the *Sapindaceae* family and/or raw milk and dairy products from grazing animals exposed to above-mentioned fruits could result in HGA contamination of mother’s breast milk. This increases risks for children, which are already the most vulnerable to HGA toxicity.

## 5. Conclusions

Grazing mares in pasture exposed to seedlings and samaras of sycamore maple present in their milk HGA and MCPA-carnitine. This unprecedented information reveals a new route of exposure of foals that could explain reported cases of AM in unweaned foals.

Contamination with HGA could represent a risk for suckling foals and to food safety by consumption of mare’s milk or potentially raw milk or dairy product from others species’ milk. This should be further investigated as in temperate European regions seasonal pasture management could decrease the risk.

## Figures and Tables

**Table 1:** Breed and age of the selected cases.

Animals		Breed	Sex	Age	Diagnosis
1	Foal 1	Paint Horse	male	58 days	Enterocolitis
	Mare 1	Paint Horse	female	8 years	Healthy cograzer
2	Foal 2	Franches-Montagnes	male	128 days	Healthy cograzer
	Mare 2	Franches-Montagnes	female	18 years	Healthy cograzer
3	Foal 3	Quarter Horse	female	37 days	*R. equi* infection
	Mare 3	Quarter Horse	female	10 years	Healthy cograzer
4	Foal 4	Zangersheide	male	171 days	Atypical myopathy
	Mare 4	Zangersheide	female	22 years	Healthy cograzer

**Table 2:** HGA and MCPA-carnitine concentration in blood and milk.

Animals		Sample Type	Sampling Date	HGA (µmol/L)	MCPA-Carnitine (nmol/L)	Creatine Kinase Activity (UI/L)
1	Foal 1	blood	24 May 2019	0.12	1.39	<20
	Mare 1	blood	28 May 2019	nd	0.35	/
		milk	28 May 2019	0.01 *	2.03	/
2	Foal 2	blood	11 November 2018	0.89	0.66	155
	Mare 2	blood	11 November 2018	0.80	0.52	/
		milk	12 November 2018	nd	0.00	/
3	Foal 3	blood	28 May 2019	0.27	0.75	<20
	Mare 3	blood	28 May 2019	0.29	8.16	/
		milk	28 May 2019	0.06 *	4.85	/
4	Foal 4	blood	19 October 2018	1.60	39.80	27,712
	Mare 4	blood	20 October 2018	0.59	2.82	/
		milk	20 October 2018	0.02 *	9.47	/

nd: not determined, * modified protocol.

## Data Availability

Restrictions apply to the availability of these data. Data was obtained from owners of horses and are available from the authors with the permission of owners of the animal involved.
